# Atom Diffusion and Evaporation of Free-Ended Amorphous SiO_x_ Nanowires: Nanocurvature Effect and Beam-Induced Athermal Activation Effect

**DOI:** 10.1186/s11671-016-1735-8

**Published:** 2016-11-23

**Authors:** Jiangbin Su, Xianfang Zhu

**Affiliations:** 1 China-Australia Joint Laboratory for Functional Nanomaterials and Physics Department, Xiamen University, Xiamen, 361005 China; 2 Experiment Center of Electronic Science and Technology, School of Mathematics and Physics, Changzhou University, Changzhou, 213164 China

**Keywords:** Athermal diffusion, Athermal evaporation, Nanocurvature, Beam-induced athermal activation, Amorphous nanowire, Uniform electron beam irradiation

## Abstract

Arresting effects of nanocurvature and electron beam-induced athermal activation on the structure changes at nanoscale of free-ended amorphous SiO_x_ nanowire were demonstrated. It was observed that under in situ uniform electron beam irradiation in transmission electron microscope, the near surface atoms at the most curved free end of the nanowire preferentially vaporized or diffused to the less curved wire sidewall. The processing resulted in an intriguing axial shrinkage and an abnormal radial expansion of the wire. It was also observed that with the beam energy deposition rate being lowered, although both the diffusion and the evaporation slowed down, the processing transferred from an evaporation-dominated status to a diffusion-dominated status. These results are crucial not only to the fundamental understanding but also to the technical controlling of the electron beam-induced structure change at nanoscale or nanoprocessing of low dimensional nanostructures.

**Electronic supplementary material:**

The online version of this article (doi:10.1186/s11671-016-1735-8) contains supplementary material, which is available to authorized users.

## Background

Transmission electron microscope (TEM) is a versatile and powerful tool with its high resolution at nanometer or even angstrom precision. It is often utilized not only for morphological or structural characterization of low dimensional nanostructures (LDNs) but also for energetic beam-induced in situ structure change or even nanoprocessing [1–4]. During the beam-induced structure change or nanoprocessing, the designed irradiation of focused, energetic electron beam (e-beam) often results in athermal atom evaporation (or ablation) [1–4], mostly on crystalline metal or semiconductor LDNs. Thus, the atom evaporation is normally regarded as the most common and important mode of mass transportation during focused e-beam irradiation of LDNs and has attracted a great deal of attention. Athermal atom diffusion is another important mode of mass transport, which was predicted [5–7] to occur during irradiation of energetic beam of lower energy deposition rate (e.g., non-focused or uniform e-beam irradiation), especially on amorphous LDNs. However, it has not attracted sufficient attention. In particular, in contrast to that of the atom evaporation, detailed manner of contribution of the atom diffusion to the structure changes of LDNs during the uniform e-beam irradiation is still unknown. In this regard, study on the diffusion of atoms and its relation with the evaporation of atoms is imperative and crucial not only to the fundamental understanding but also to the precise and flexible controlling of the e-beam-induced structure change or nanoprocessing.

On the other hand, people normally resort to the existing knock-on mechanism [8] to explain or predict the energetic (ion or electron) beam-induced nanophenomena including beam-induced dynamic atomic defect creation and annihilation and atom transport processes on LDNs. However, it has been demonstrated that knock-on mechanism cannot offer a full explanation for the experimentally observed beam-induced nanophenomena, especially for these such as the plastic flow or wetting of carbon nanotube [9] and amorphous SiO_x_ nanowire [7], or the shrinking nanocavity along with its cavity-near-surface preferential amorphization [5, 10, 11] as observed earlier in silicon during energetic beam irradiation. This is because the existing theories such as the knock-on mechanism were at the first place built on consideration of the nature of equilibrium, symmetry, periodicity, and linearity of bulk crystalline structure or its approximation, whereas the beam (including electron, ion, and photon beams)-induced nanophenomena are intrinsically of non-equilibrium, amorphous, and non-linear nature. The previous research [5, 9–12] has demonstrated that our proposed nanocurvature or nanosize effect [5, 13] and energetic beam-induced athermal activation or nanotime (soft mode and instability of atomic vibration) effect [5, 6] could well explain the above beam-induced nanophenomena and widespreadly control the beam-induced structure changes and nanoprocessing of LDNs. Thus, it has been further asserted that both nanocurvature effect and beam-induced athermal activation effect would control as well dynamic behaviors of atom diffusion and evaporation of one-dimensional (1D) nanowire during uniform e-beam irradiation. However, there is no report found to demonstrate the effects of nanocurvature and beam-induced athermal activation on the structure changes of nanowires during uniform e-beam irradiation (so far, the only report we can found dealing with the above effects on e-beam-induced structure changes or nanoprocessing of nanowires is in reference [7] but under focused e-beam irradiation). Thus, the nanocurvature effect and beam-induced athermal activation effect underlying the dynamics of atom diffusion and evaporation in nanowires under uniform e-beam irradiation remain unclear.

With the above considerations, in this paper, we specifically study the nanocurvature effect and the beam-induced athermal activation effect on the diffusion and evaporation of atoms on free-ended amorphous SiO_x_ nanowires under uniform e-beam irradiation in TEM. We observed a preferential surface evaporation at the free end of the wire and a directional surface diffusion from the free end to the wire sidewall which were driven by nanocurvature effect. Such preferential evaporation and directional diffusion of atoms resulted in an intriguing axial shrinkage and an abnormal radial expansion of the wire. It was also observed that with the beam energy deposition rate being lowered, although both the diffusion and the evaporation slowed down, the processing transferred from an evaporation-dominated status to a diffusion-dominated status.

## Method

The amorphous SiO_x_ nanowires were grown by our improved chemical vapor deposition set-up where *x* is determined to be 2.3 [14]. They were well-dispersed in ethanol and then deposited onto holey carbon film on Cu grids for TEM studies. The TEM specimens as prepared were subsequently irradiated at room temperature, and the structure evolution of SiO_x_ nanowires were in situ observed via a field-emission Tecnai F30 TEM operated at 300 kV. The irradiation was always targeted on single wire segment with one free end protruding into the open space of the holes in the carbon film of microscopy grid. Also, the wire segment with one free end was an ideal candidate for the investigation of nanocurvature effect due to the non-uniform distribution of nanocurvature on the wire surface. In each irradiation, the current density at the specimen was kept at about 1 A/cm^2^ (flux 6.25 × 10^4^ nm^−2^ s^−1^) or 10 A/cm^2^ (flux 6.25 × 10^5^ nm^−2^ s^−1^), which was uniform over an area larger than the zone observed. Such two designed sets of irradiation with different current densities made possible the study on beam-induced athermal activation effect. During the observation or taking a picture, the beam was spread to an around 100 times weaker intensity so that the corresponding irradiation effect can be minimized to a negligible degree and at the same time the image contrast can also be improved. Also note that during the electron irradiation, the beam was expected to heat the specimen by no more than a few degrees [4, 7, 8] due to its extremely large ratio of surface to volume, and the dominant irradiation effect should be athermal. Therefore, it could be considered that the irradiated nanowire essentially remained at room temperature throughout the irradiation duration.

## Results and Discussion

**Fig. 1:**
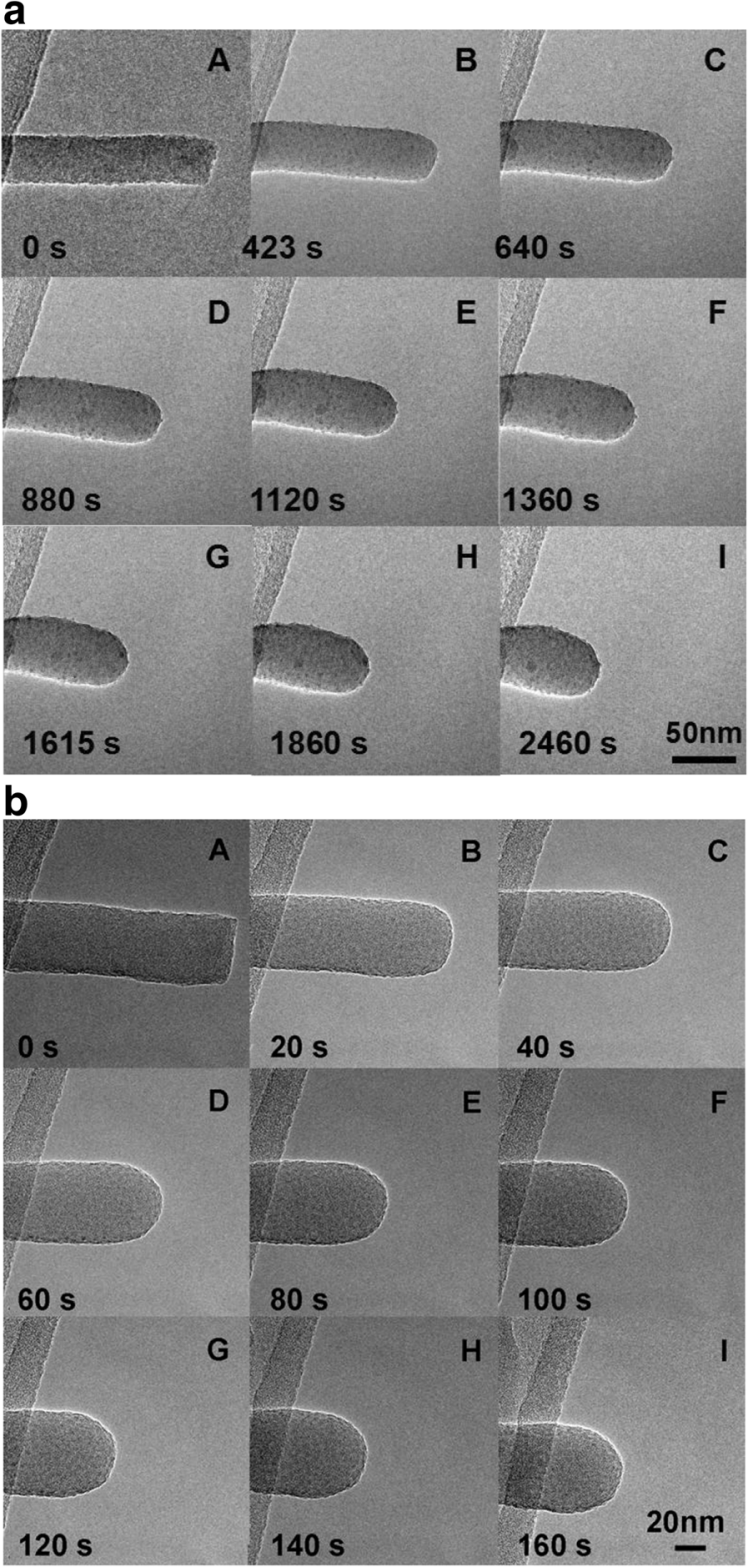
Sequences of in situ TEM micrographs showing the typical structural evolution of the free-ended amorphous SiO_x_ nanowires during uniform irradiation of e-beam with different current densities respectively at **a** 1 A/cm^2^ and **b** 10 A/cm^2^

Figure 1 shows the typical in situ structural evolution of amorphous SiO_x_ nanowire segments with single free ends during uniform irradiations of e-beam with different current densities respectively at (a) 1 A/cm^2^ and (b) 10 A/cm^2^. Before the irradiation, both the SiO_x_ nanowires as shown in A of Fig. 1a, b represented a well-defined straight, columned segment of wire with clean and smooth surface, uniform diameter, and sharp cut edge at the free end. After an initial period of irradiation (respectively at 640 s in Fig. 1a and at 40 s in Fig. 1b), the sharp cut edges became round, and the free ends showed hemisphere shapes. Meanwhile, Fig. 1a, b demonstrated a preferential axial shrinkage from the free end and an abnormal increase of wire diameter which were also observed throughout the remaining irradiation. Normally, such increase of wire diameter or radial growth was limited within the wire segment near the free end and thus cannot be uniform along the entire wire or even the irradiated wire segment. Furthermore, both the axial shrinking and the radial increasing in Fig. 1b were obviously much faster than those in Fig. 1a with the increased beam current density (for axial shrinking rate 5.7 × 10^−1^ vs. 3.5 × 10^−2^ nm/s; for radial increasing rate 6.3 × 10^−2^ vs. 6.2 × 10^−3^ nm/s, see Additional file 1: Supporting Information 1). The similar irradiations on different wire segments under different beam current densities were repeated several times, and the structural evolution were essentially the same.

Different from the focused irradiation cases [1–4] where only atom evaporation (or ablation) dominates, athermal surface atom diffusion along with the surface atom evaporation is expected to occur during the above uniform e-beam irradiation. For the surface atom diffusion, atoms diffusing along the wire surface from one location to another could lead to a re-distribution of wire atoms, while for the surface atom evaporation, atoms escaping from the wire surface would result in a pure loss of wire materials. In this work, as shown in Fig. 1, the free end with sharp cut edge represented a strong and general tendency of being hemisphere-shaped or rounded. Moreover, the wire universally showed a preferential axial shrinkage from the free end no matter whether it is with sharp cut edge or of hemisphere shape. Similar axial shrinkages preferentially from the free end were also observed in carbon nanotubes [15, 16] as a universal phenomenon. All of these indicated that the nanocurvature effect dominated or governed the dynamics of surface diffusion and evaporation of atoms. On the other hand, as shown in Fig. 1 and Additional file 1: Figure S1, the fact that different current densities resulted in different wire shapes and structural evolution rates demonstrated that the beam-induced athermal activation effect would also greatly influence the surface diffusion and evaporation of atoms.

For the nanocurvature effect on a nanowire, we can suppose that, similar to the nanoparticle case [5, 13], when the diameter of a nanowire approaches its atomic bond length, a positive nanocurvature on the wire surface will become appreciable. The nanocurvature would cause a substantial distortion or change to the electron cloud or the bonding structure of near-surface atoms and a strong deviation of atoms from their equilibrium state. Consequently, an additional tensile stress would build up on the wire surface and dramatically increase the surface energy. This dramatically increased surface energy would lower the energy barrier for the atoms to migrate or escape thus causing intrinsic structure instability in the nanowire.

**Fig. 2:**
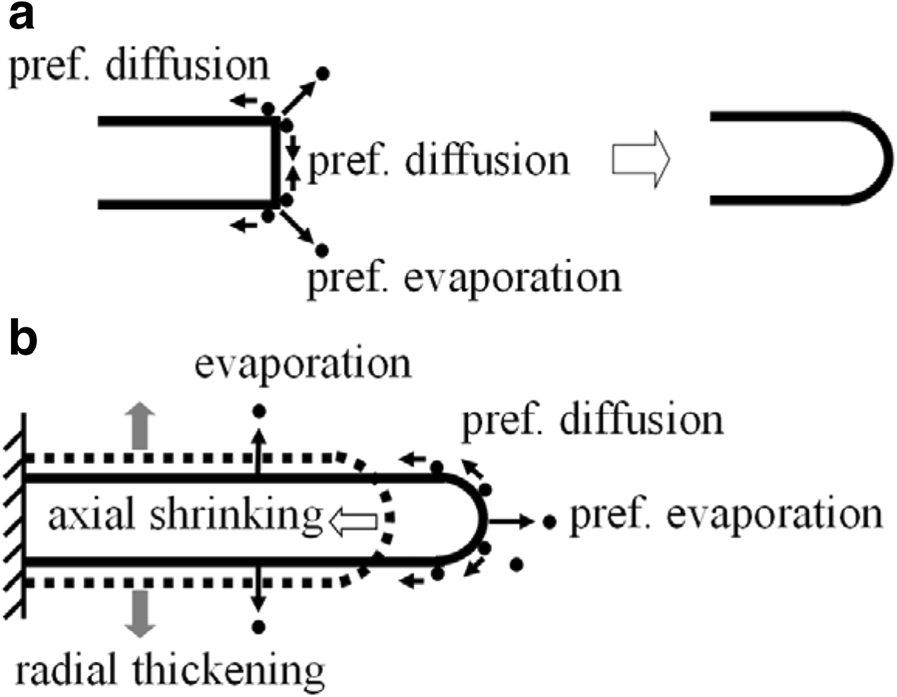
Schematic illustration showing the directional surface diffusion and the preferential surface evaporation of atoms driven by nanocurvature effect: **a** free end with cut sharp edge case and **b** free end of hemisphere shape case

As illustrated in Fig. 2a, the starting nanowire shows a sharp cut edge at the free end, which is the most curved location relative to the cylindrical sidewall and the plane bottom. According to the nanocurvature effect of a nanowire, the most curved sharp cut edge is the most unstable location with the highest surface energy and the lowest energy barrier. So, under the activation of e-beam irradiation, the atoms at the sharp cut edge would migrate to the wire sidewall or bottom, or escape from the wire surface preferentially (see Fig. 2a) to minimize the total energy of the nanowire. As a consequence, the free end became round and smooth with a hemisphere shape of uniform positive surface nanocurvature finally (see A–C of Figs. 1a, b and 2a).

In the subsequent irradiation, the free end of the wire kept the hemisphere shape with a larger nanocurvature than that on the wire sidewall. Driven by the nanocurvature effect, as shown in Fig. 2b, the atoms at the free end would prefer to either diffuse (migrate) to the wire sidewall or escape from the wire surface, and thus, a preferential axial shrinkage of nanowire occurred. Meanwhile, the diffused atoms would further accumulate on the wire sidewall. Although atoms on the wire sidewall might also vapor under irradiation, the hypothetical resulting tendency of radial shrinkage was overcome by the incoming diffusion of atoms from the free end. As a consequence, an abnormal radial thickening which was in contradiction with the existing knock-on mechanism [8] was observed instead of a hypothetical radial shrinking. Also note that the atom diffusion starts from the free end and there is an increasing distance for the atoms to overcome or migrate to the other end. The atom evaporation and diffusion or the extent of radial growth would thus be limited by the beam energy, current density, and the irradiation duration, and so far, there was no detectable effect as found from the carbon film. As a consequence, only the segment near the free end exhibited a visible radial growth especially under a limited current density or within a short irradiation duration. Similar to the case of hemisphere-shaped free end, as shown in A–C of Fig. 1a, b and Additional file 1: Figure S1, the starting nanowire with a highly curved sharp edge at the free end also showed a preferential axial shrinkage and an abnormal increase of wire diameter. It once again demonstrates that the structure changes of a nanowire and the underlying dynamics of surface diffusion and evaporation of atoms are governed by the intrinsic nanocurvature effect of the nanowire. That is, surface atoms would tend to diffuse directionally from one location with a relatively higher nanocurvature to another with a relatively lower nanocurvature or vaporize preferentially at the location with a relatively higher nanocurvature.

Although the positive nanocurvature can lower the energy barrier of atoms and thus causes structural instability, a further assistance from external excitation such as energetic beam irradiation is still needed to realize finally the ultrafast mass transportation. In the present case of energetic e-beam irradiation in TEM, we can assume that when the beam energy deposition rate (detailed in the following) becomes very fast, the atoms would not have enough time to convert the deposited beam energy to their thermal vibration energy via inelastic interaction processes. Thus, the mode of atom thermal vibrations would be softened or the atom vibrations would lose stability, which can further suppress the energy barrier for the atoms to migrate or escape and finally make the structure changes kinetically possible. This phenomenon was called beam-induced athermal activation effect or nanotime effect in a broad sense [5, 6].

The above mentioned beam energy deposition rate on specimen is defined as the beam energy deposited per volume of specimen per duration of irradiation. After a simple calculation, we could easily get the beam energy deposition rate *v*_*E*_

1 $$ {v}_E=\underset{\varDelta V\to 0}{ \lim}\left|\varDelta E/\left(\varDelta t\cdot \varDelta V\right)\right|=jU/h $$

where *ΔE* is the beam energy deposited on specimen, *ΔV* is the specimen volume irradiated, *Δt* is the duration of irradiation, *j* is the irradiation current density, *U* is the accelerating voltage, and *h* is the local thickness of specimen along the direction of incident beam. According to Eq. (1), the beam energy deposition rate is proportional to the irradiation current density. So, in this work, comparing with Fig. 1a, a higher current density in Fig. 1b would result in a higher beam energy deposition rate and thus cause more pronounced structural instabilities for the atoms to migrate or escape more easily.

**Fig. 3:**
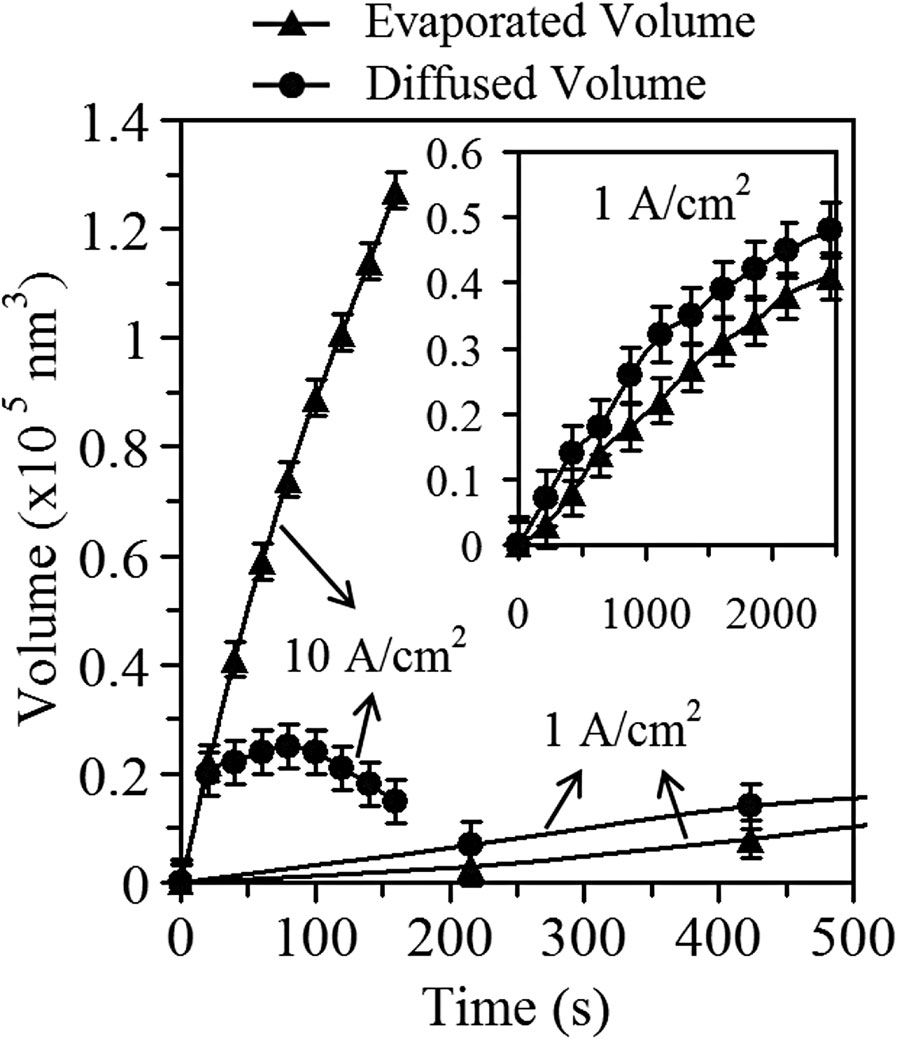
Evolution of evaporated or diffused volume with irradiation time under different current densities. The inset with the same axis units shows the whole evolution curves of evaporated and diffused volumes under current density of 1 A/cm^2^

To reveal the beam-induced athermal activation effect on the atom diffusion and evaporation, we studied the real-time diffused and evaporated volumes of nanowires under different current densities (the calculation method can be found in Additional file 1: Supporting Information 2). As shown in Fig. 3, the evolution of the evaporated volume and diffused volume under different current densities were plotted with irradiation time. It was found that with the decrease of current density from 10 to 1 A/cm^2^, the average evolution rates of evaporated volume and diffused volume decrease from 7.9 × 10^2^ to 1.7 × 10^1^ nm^3^/s and from 9.4 × 10^1^ to 2.0 × 10^1^ nm^3^/s, respectively (see Additional file 1: Supporting Information 3). For a further confirmation, we repeated the experiments by changing the current density and got the same conclusions. It thus demonstrates that with the current density or energy deposition rate being lowered, both the diffusion and the evaporation slowed down and vice versa.

In the following, we further compared the contribution of atom diffusion with that of atom evaporation to the structure changes under different current densities. As also shown in Fig. 3, the diffused volume was always larger than the evaporated volume at a lower current density of 1 A/cm^2^. What is more, as we have mentioned above, the real diffused volumes should be larger than the as-calculated values due to a further evaporation of the previously diffused atoms to the wire sidewall. Both demonstrate that at a relatively lower current density such as 1 A/cm^2^, the diffusion of atoms contributes more to the structural changes or dominates over the evaporation of atoms. However, when the current density was increased to 10 A/cm^2^, as shown in Fig. 3, the evaporated volume was larger than the diffused volume on the contrary. Although the real diffused volume was still unclear, we could suppose the extreme case that when the e-beam was highly focused with current density high enough, it has been experimentally demonstrated that only evaporation of atoms could be observed [1–4]. Accordingly, we could conclude that at a relatively higher current density such as 10 A/cm^2^, the evaporation of atoms plays a more important role in the structure changes or dominates over the diffusion of atoms. After the irradiation, the different resulting wire morphologies of the wire sidewall in Fig. 1a showed a hump-like shape, while the wire segment in Fig. 1b seemed to keep its cylinder sidewall shape that can well confirm the above beam current density-dependent contribution mechanisms of atom diffusion and evaporation. That is, the accumulation effect of diffused atoms on the wire sidewall was especially more pronounced in the case of Fig. 1a, where under the irradiation with a lower beam current density, the atom diffusion obviously dominated over the atom evaporation to a much higher degree than in the case of Fig. 1b.

## Conclusions

In this paper, the arresting effects of nanocurvature and e-beam-induced athermal activation on the structure changes of free-ended amorphous SiO_x_ nanowire were demonstrated. It was observed that under in situ uniform e-beam irradiation in TEM, the near surface atoms at the most curved free end of the nanowire preferentially vaporized or diffused to the less curved wire sidewall. The processing resulted in an intriguing axial shrinkage and an abnormal radial expansion of the wire. It was also observed that with the beam energy deposition rate being lowered, although both the diffusion and the evaporation slowed down, the processing transferred from an evaporation-dominated status to a diffusion-dominated status. The above processing involved selective, directional, and ultrafast atom transportations and cannot be adequately explained by the existing knock-on mechanism. However, it can be well interpreted by combination of the nanocurvature effect of amorphous SiO_x_ nanowires and the athermal activation effect of uniform e-beam irradiation. It is worth noting that the nanocurvature effect and the beam-induced athermal activation effect for the SiO_x_ nanowire structure changes would be intrinsically of non-equilibrium, amorphous, and non-linear nature. Such a study on these effects is crucial not only to the fundamental understanding but also to the technical controlling of the e-beam-induced structure change or nanoprocessing of LDNs.

## Additional file

Additional file 1: Supporting Information 1: The calculation results of the length and diameter changes under different current densities. Supporting Information 2: The calculation methods of the evaporated and diffused volumes. Supporting Information 3: The calculation results of the evaporated and diffused volumes under different current densities. (DOCX 112 kb).
