# Supplementary material for: Atom Diffusion and Evaporation of Free-Ended Amorphous SiOx Nanowires: Nanocurvature Effect and Beam-Induced Athermal Activation Effect
Source: Nanoscale Res Lett. 2016 Nov 23;11:514. doi: 10.1186/s11671-016-1735-8 (PMC5120135; doi:10.1186/s11671-016-1735-8)
Supplement: Additional file 1: — Supporting Information 1: The calculation results of the length and diameter changes under different current densities. Supporting Information 2: The calculation methods of the evaporated and diffused volumes. Supporting Information 3: The calculation results of the evaporated and diffused volumes under different current densities. (DOCX 112 kb). [file 11671_2016_1735_MOESM1_ESM.docx]

**Atom diffusion and evaporation of free-ended amorphous SiO_x_ nanowires: nanocurvature effect and beam-induced athermal activation effect**

Jiangbin Su,^1,2^ and Xianfang Zhu^1,*^

*^1^China-Australia Joint Laboratory for Functional Nanomaterials and Physics Department, Xiamen University, Xiamen 361005, China*

*^2^Experiment Center of Electronic Science and Technology, School of Mathematics and Physics, Changzhou University, Changzhou 213164, China*

*^*^Corresponding author. E-mail: [zhux@xmu.edu.cn](mailto:zhux@xmu.edu.cn)*

**Supporting Information 1**

As illustrated in Fig. S1(a), under the irradiation with the current density of 1 A/cm^2^, the wire length shrunk slowly from 159.7 nm to 73.9 nm within 2460 s with an average axial shrinking rate of 3.5×10^-2^ nm/s whereas under the irradiation with the current density of 10 A/cm^2^, the wire length quickly shrunk from 137.9 nm to 47.4 nm just within 160 s with an average axial shrinking rate of 5.7×10^-1^ nm/s. As shown in Fig. S1(b), under the irradiation with the current density of 1 A/cm^2^, the wire diameter increased slowly from 36.4 nm to 51.7 nm within 2460 s with an average radial thickening rate of 6.2×10^-3^ nm/s; while under the irradiation with the current density of 10 A/cm^2^, the wire diameter increased quickly from 44.8 nm to 54.8 nm within 160 s with an average radial thickening rate of 6.3×10^-2^ nm/s. By contrast, when the irradiation current density was elevated by one order of magnitude (from 1 A/cm^2^ to 10 A/cm^2^), the structural evolution rates such as the average axial shrinking rate and the average radial thickening rate were both magnified by about ten times or even more.


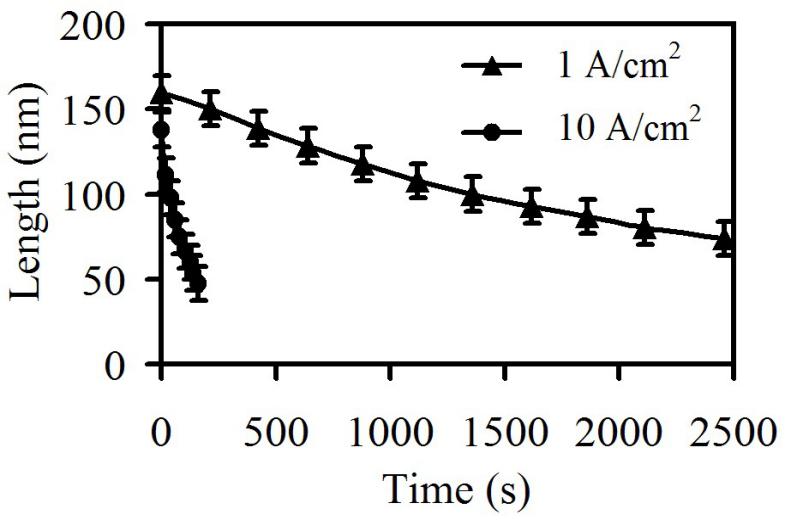


(a)


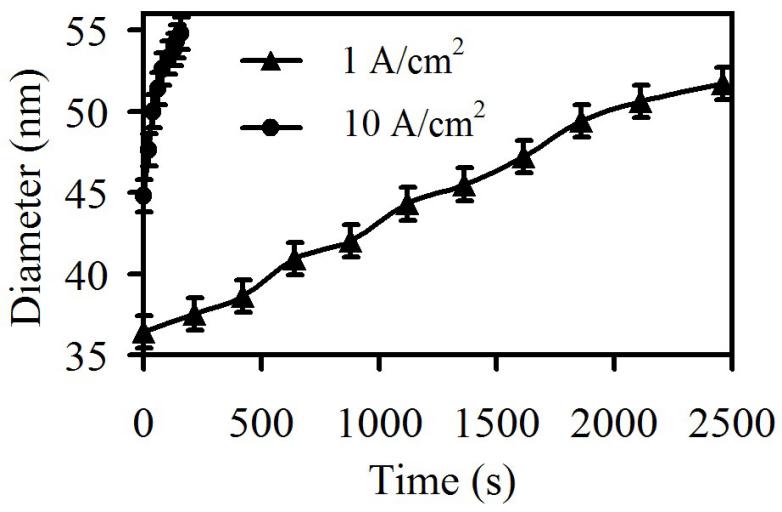


(b)

**Fig. S1** Length (a) and diameter (b) evolutions of the SiO_x_ nanowires with irradiation time as observed in Fig. 1.

**Supporting Information 2**


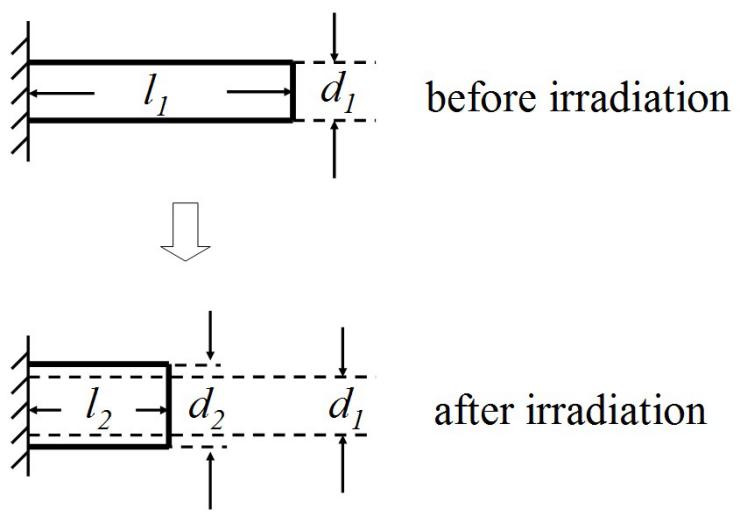


**Fig. S2** Schematic illustration showing the length and diameter changes of a nanowire segment after irradiation.

For simplicity, we made the following two assumptions: (i) the net loss of materials is owing to the atom evaporation; (ii) the net radial thickening is caused by the atom diffusion. Accordingly, as illustrated in Fig. S2, we could obtain the evaporated volume $V_{\text{evap}}$ and the diffused volume $V_{\text{diff}}$ respectively

$V_{\text{evap}}=V_{l_{1}d_{1}}-V_{l_{2}d_{2}}$ (S1)

$V_{\text{diff}}=V_{l_{2}d_{2}}-V_{l_{2}d_{1}}$ (S2)

where $V_{l_{1}d_{1}}$ is the volume of wire segment before irradiation with length $l_{1}$ and diameter $d_{1}$, $V_{l_{2}d_{2}}$ is the volume of wire segment after irradiation with length $l_{2}$ and diameter $d_{2}$, $V_{l_{2}d_{1}}$ is the volume of a fabled wire segment with length $l_{2}$ and diameter $d_{1}$. The volume of wire segment is approximately calculated as a sum up of volumes of many smaller regular geometries such as cylinders, circular truncated cones, hemisphere, etc., which further divide the wire segment but become calculable. Here, we should also note that the real diffused volumes would be larger or even much larger than the as-calculated results by Eq. (S2). This is because part of the diffused atoms which aggregate on the wire sidewall or at the free end would further be evaporated and lost in the subsequent irradiation. In spite of this, it still can provide some references and suggestions which will be discussed in the following.

**Supporting Information 3**

(i) For the current density of 1 A/cm^2^, the evaporated volume increased by 0.41×10^5^ nm^3^ within 2460 s with an average rate of 1.7×10^1^ nm^3^/s; while for the current density of 10 A/cm^2^, the evaporated volume increased by 1.27×10^5^ nm^3^ within 160 s with an average rate of 7.9×10^2^ nm^3^/s. It indicates that the evaporation rate of atoms increases with irradiation current density or beam-induced athermal activation effect and vice versa.

(ii) For the current density of 1 A/cm^2^, the diffused volume increased by 0.48×10^5^ nm^3^ within 2460 s with an average rate of 2.0×10^1^ nm^3^/s; while for the current density of 10 A/cm^2^, there were three different periods for the evolution of diffused volume: from 0 s to 20 s, it increased quickly with an average rate of 1.0×10^3^ nm^3^/s; from 20 s to 100 s, it increased slowly with an average rate of 5.0×10^1^ nm^3^/s; from 100 s to 160 s, it decreased with a negative average rate of –1.5×10^2^ nm^3^/s. One of the most important reasons for the quick slowing down or even decrease is the diffused atoms on the wire surface were further evaporated in the subsequent irradiation especially in the case of high current density with a fast evaporation rate. Even so, the average diffusion rate throughout the irradiation at current density of 10 A/cm^2^ was 9.4×10^1^ nm^3^/s, which was still much larger than that at current density of 1 A/cm^2^ (2.0×10^1^ nm^3^/s). Furthermore, as have shown in Fig. S1, the radial thickening caused by atom diffusion was much faster at current density of 10 A/cm^2^ than that at current density of 1 A/cm^2^. All of these indicate that the diffusion rate of atoms increases with current density or beam-induced athermal activation effect and vice versa.
